# Heavy Metal Toxicity in Cereals: Uptake Mechanisms, Physiological Impacts, and Mitigation Strategies

**DOI:** 10.3390/toxics13121074

**Published:** 2025-12-13

**Authors:** Kashish Singh, Chandranandani Negi, Ajay Kumar, Navaneet Chaturvedi, Pritesh Vyas

**Affiliations:** 1 School of Applied and Life Sciences, Uttaranchal University, Dehradun 248007, Uttarakhand, India; 2 Department of Science & Technology, Jayoti Vidyapeeth Women’s University, Jaipur 303122, Rajasthan, India; 3 Amity Institute of Biotechnology, Amity University, Noida 201313, Uttar Pradesh, India

**Keywords:** wheat, heavy metals, cadmium, mercury, lead, soil, mitigation

## Abstract

Heavy metal (HM) toxicity is one of the growing concerns, posing a significant threat to food security. Its trace presence in the food is one of the major reasons for considering it as a threat, which makes it potentially dangerous and a widespread concern. Post-Green Revolution, production and, thereafter, nutrition were given attention, but in the present decade, HM toxicity, its uptake, physiological impact, and mitigation are the major research interests. Cereals are potent food materials that hold a huge consumer market. The presence of these HMs in cereals in higher concentrations than the standard makes them toxic to consume and has caused a global crisis. This toxicity is silently impacting the genetic homeostasis of the ecosystem and, most importantly, the human body. Frequent occurrence of carcinoma, genetic disorders, and phenotypic deformities is the major outcome of this contamination. Its presence in the soil threatens the microflora and fauna of the ecosystem, thus interrupting the complete natural process of energy exchange between the system and the surroundings. It is therefore of the utmost importance to understand the uptake and physiological mobilization of these HMs and their mitigation strategies for a sustainable & green ecosystem. The present review comprehensively analyzes the biological and ecological losses due to these HMs and their mitigation in plants with special reference to cereals.

## 1. Introduction

One of the most significant environmental problems of the 21st century is heavy metal contamination, which is mostly brought on by fast industrialization, mining, intensified agricultural activities, and urbanization [1]. The background load increases through natural sources, such as weathering of rock and volcanic activity, while anthropogenic activities, including the application of fertilizer and pesticides, wastewater irrigation, livestock manure, and emissions of industrial processes, result in high levels of toxic metal enrichment in soils [2].

Heavy metals (HMs), which include the extremely hazardous elements such as cadmium (Cd), arsenic (As), lead (Pb), mercury (Hg), and chromium (Cr) [3,4], are inorganic pollutants with high density (>5 g/cm^3^) [5], high atomic weight [6], and most importantly, an exceptional capability of bioaccumulation. These contaminants can linger in the soil ecosystem for several decades, reducing fertility, disturbing microbial communities, and endangering the sustainability of agricultural production because they are not biodegradable like other organic pollutants [7]. The National Toxicology Program, WHO (World Health Organization), and the IARC (International Agency for Research on Cancer) [8] have ranked some heavy metals in food, especially Cr and Cd, as group 1 carcinogens because of the risk they pose to human health [9,10].

HMs immediately act on plant roots upon addition to soil, representing the main entrance into the food chain. Upon uptake, metals may be immobilized in root tissue and transported via phyto-extraction, phytostabilization, or rhizo-filtration, respectively [11]. This transport leads to serious consequences: heavy metals act by disrupting photosynthesis [12], respiration, and enzymatic processes, and, via their excessive accumulation, oxidative stress and production of ROS [12] can be induced to bring about growth, reproductive potential, and productivity inhibition in plants [13].

Besides the issues of agriculture, heavy metal absorption has adverse effects on the integrity of the environment and human health. Toxic metals are harmful to both humans and animals since they are deposited in the tissue of edible plants and enter the food web [14,15]. Upon entry into this cycle, they take part in biomagnification at successively higher trophic degrees [16]. Therefore, long-term exposure to contaminated food sources has been linked with various health disorders, including delayed brain development, several types of cancer, cardiovascular diseases, and renal failure [17,18]. The influence of heavy metal poisoning also extends to the economic and societal sectors. However, the issue is more prevalent in many developing nations, in part because of the aforementioned factors and possibly in part because of a lack of knowledge regarding the harmful effects of these elements on crop health as well as human health [19,20]. Thus, there is a critical need for a holistic understanding of the sources, flow, and impacts of heavy metals in the soil–plant-human continuum.

Overall, in light of the highlighted ecological dangers, this review covers information on sources of HMs and their uptake and transport mechanisms. It also discusses the eventual consequences of HMs on the health of soil and their impacts on plants. In addition, it focuses on a holistic mitigation strategy to support the protection and creation of sustainable agriculture. The novelty of this study is to provide an in-depth review of information about how heavy metals originate from different sources, accumulate in soil and plants, and eventually reach humans. Additionally, the toxicity of HMs in soil and plants has been discussed, followed by a critical analysis of strategies that are needed in modern times to attain secure and sustainable agriculture.

## 2. Origin and Distribution of the HMs in Agroecosystems

The two main categories of heavy metal sources identified by the scientists are anthropogenic and natural. Agricultural soil is one significant natural resource that is easily polluted by both natural and anthropogenic factors. Anthropogenic sources include mining, industry, agriculture, and household wastewater, whereas natural sources include sedimentary rock, weathering of rock that bears metals by atmospheric deposition and rainwater, and volcanic eruptions [21,22,23,24]. Nevertheless, the continuous addition of heavy metals to cropland can produce soil that is too toxic to sustain plant development and productivity, regardless of the source of contamination [24]. The subsequent subsections mainly examine how probable agricultural activities can contaminate farmlands with HMs, among other factors.

### 2.1. Geogenic Sources and Natural Input of HMs

Sedimentary and igneous rocks are thought to be the most prevalent natural sources of HMs like As, Pb, F, and Zn. The type of rock and the ecological parameters of the surrounding area can be used to determine the concentration ranges (ppm) of heavy metals [12,25]. For example, Cd has a range (ppm) of 0.006–0.6 in basaltic igneous, 0.003–0.18 in granite igneous, and <0.3–8.4 in black shales; Pb has a range of 30–160 in basaltic igneous, 4–30 in granite igneous, and 20–200 in black shales; and Zn has a range of 2–18 in basaltic igneous, 6–30 in granite igneous, 7–150 in black shales, and 2–41 in sandstones [12,26]. Furthermore, aside from river sediments, soil formation is thought to be one of the primary causes of heavy metal buildup.

### 2.2. Anthropogenic Sources of HMs

Mining, wastewater, industries, and agriculture are all categorized under anthropogenic sources of HMs, especially cadmium, chromium, and copper. For example, smelting results in the release of As, Cu, Pb, and Zn, and pesticides result in the release of As, greatly increasing the concentration of HMs in the ecosystem [12,27]. Furthermore, routine human endeavors like farming, industrial operations, fossil fuel combustion, mining, and manufacturing disrupt the biosphere’s equilibrium [12,28].

### 2.3. Agricultural Sources

A number of contaminants, including agricultural toxins—also referred to as biotic and abiotic consequences of farming practices—typically have an impact on ecosystems associated with agriculture. The surrounding agroecosystems are typically contaminated and degraded by these pollutants [29]. The most prevalent agricultural sources of HMs are fertilizers, insecticides, sewage sludge [30], etc.

#### 2.3.1. Fertilizers as Heavy Metal Inputs

They increase the amount of organic matter in the soil and provide various nutrients that plants need to develop and grow. However, HMs in soil can be generated by fertilizers, which include both organic and inorganic components [12]. Chemical fertilizers, particularly inorganic ones, are critical for increasing crop output because they provide key macronutrients, including potassium (K), phosphorous (P), and nitrogen (N). Phosphorus, which has extensive use in the manufacturing of fertilizers, leads to the accumulation of heavy metals in soils [31], and the largest amounts of heavy metal (HM) pollutants, such as Cd, Co, Cu, Pb, Zn, Cr, and Ni, are found in phosphorus (P) fertilizers [18,32,33,34]. The water-insoluble phosphatic fertilizers result in the production of phosphate rocks that precipitate metals in the form of metal phosphates [12,35]. The excessive and repetitive use of fertilizers leads to the fixation of metals like Cu, Zn, and Cd, making the soils barren and less productive for crops [36,37].

#### 2.3.2. Pesticides as a Source of Soil Contamination

Since they prevent an estimated 40 percent of the world’s food production from declining, pesticides are essential to contemporary agriculture [16], averting around one-third of all agricultural losses worldwide [38]. The current estimate of the world’s yearly pesticide usage is 2 million tons, of which 47.5% are herbicides, 29.5% are insecticides, 17.5% are fungicides, and 5.5% are additional kinds [12,39,40]. These products include harmful organic or inorganic substances. Copper sulphate (Bordeaux mixture), lead arsenate, and copper acetoarsenite were among the historically used insecticides that introduced heavy metals (HMs) like Hg, Cr, As, Cu, Pb, and Zn to soils [18,41]. Further, it has been discovered that HM impurities such as Cd, Hg, As, Cu, Zn, and Pb are present in many contemporary pesticide formulations, either inadvertently added during manufacture or purposefully added in nano-form to increase performance [18,42,43]. However, the extent of pesticide-induced contamination mostly depends on chemical persistence, application frequency, and soil properties, making their long-term ecological and agronomic implications very varied and typically underestimated.

#### 2.3.3. Compost and Livestock Manure as Contaminant Carrier

Poultry, cattle, and pigs are the primary sources of livestock manures, which are used as organic fertilizers but also include significant amounts of heavy metals (HMs) such as Ni, Cr, As, Cu, Pb, Zn, Cd, and Hg [18,44,45,46]. Growth-promoting minerals and organic arsenicals are among the commercial feed additives that are primarily responsible for these pollutants [42,44,45]. These metals are excreted in manure by animals because they are unable to metabolize them, and because they do not break down, they remain after composting [47]. Soil accumulation of harmful heavy metals (HM) might result from frequent applications of compost or manure, endangering crop productivity and growth [48,49].

#### 2.3.4. Irrigation with Contaminated Water

Another major source of HM penetration into soils, especially in developing nations, is irrigation using contaminated groundwater or surface water [50,51,52,53]. The pollutants come from anthropogenic sources such as industrial waste discharge and agricultural runoff, as well as natural processes like weathering and air deposition [53,54,55]. Metals including Cd, As, Hg, Ni, Cr, Zn, and Cu are carried into the soils via runoff and leaching, which ultimately reduces agricultural production and soil quality [56]. The degree of contamination effects in irrigation water is determined by factors such as pH, metal solubility, and redox potential.

## 3. Impact of Heavy Metal Accumulation on Soil Health

Even though heavy metals are believed to be part of the soil, high concentrations of these metals can have detrimental effects on both the soil and plants. As a result, they are regarded as toxicants [12,57]. The lack of macronutrient availability and the acidity of the soil are two of the main problems associated with the buildup of heavy metal toxicity [12].

Among the heavy metals, cadmium accumulation in soil is a pervasive issue with the rapid industrial development, economic revolution, and current agricultural technologies [12]. Generally, the two most prevalent factors affecting Cd accumulation are soil pH and organic matter content. With an increase in the decline of soil pH, Cd bioavailability increased, reflecting a disturbance in the properties of soil [12]. A study by Raisei and Sadeghi [28] focused on the interactive effects of salinity and Cd on soil microorganisms and enzymatic activity. According to their findings, salinity, and Cd act synergistically to adversely affect the properties of soil, as well as affect the microorganisms that are useful for soil health by inhibiting their activity and altering the physiochemical traits [26,29].

Pb poisoning mainly affects *Eisenia fetida*, which results in earthworm mortality [12]. In the findings of Kumar et al. [58], a negative association between soil pH and Pb solubility was discovered, suggesting that Pb buildup in the soil results in a flaw in the plant absorption mechanism. Pb shows high toxic effects towards soil fertility and microbial activity even at low concentrations [59,60]. According to [61], Pb also has an impact on the soil’s humic acid concentration and sorption capability. Pb’s and Cd’s individual and combined impacts on soil microbial populations and some enzyme activities were investigated by Khan et al. [62], and the results demonstrated that the contamination had a significant impact on the microbial communities [12]. Thus, Pb not only affects plants but also shows detrimental effects on the soil’s microflora and fauna.

Cu, being an important component of the soil, is also a crucial micronutrient required by the plants [12]. An instance of poisoning associated with Cu toxicity results in a flaw in any system where the levels are higher than supra-optimal [63]. Cu toxicity has been shown in numerous studies to considerably reduce soil microbial activity. Additionally, Cu poisoning can denature microbial proteins and damage cell membranes. Cu’s harmful effects on soil microorganisms and microbial biomasses were investigated by Wang et al. [64]. The organism most severely impacted was bacteria, followed by actinomycetes and fungi [12,64].

Zinc is another crucial microelement that supports plant development hormones and proteins [65]. It actively participates in the metabolic physiological processes of plants due to its involvement in sugar absorption. However, because zinc poisoning negatively impacts soil microorganisms that enhance soil fertility and structure, it is a hazard [66]. It also affects active sites of soil enzymes as it replaces some cations that are required for cell function [67]. The ultimate result of the accumulation of HMs includes reduced fertility, lower biological activity, and degradation of soil, which affects the ultimate capacity of soil to support crop development healthily and sustainably [12,18]. Zinc and other metals disrupt the soil’s ecological homeostasis and also inhibit the enzyme activities of the microbial community.

## 4. Heavy Metal Dynamics in Cereals

**Figure 1:**
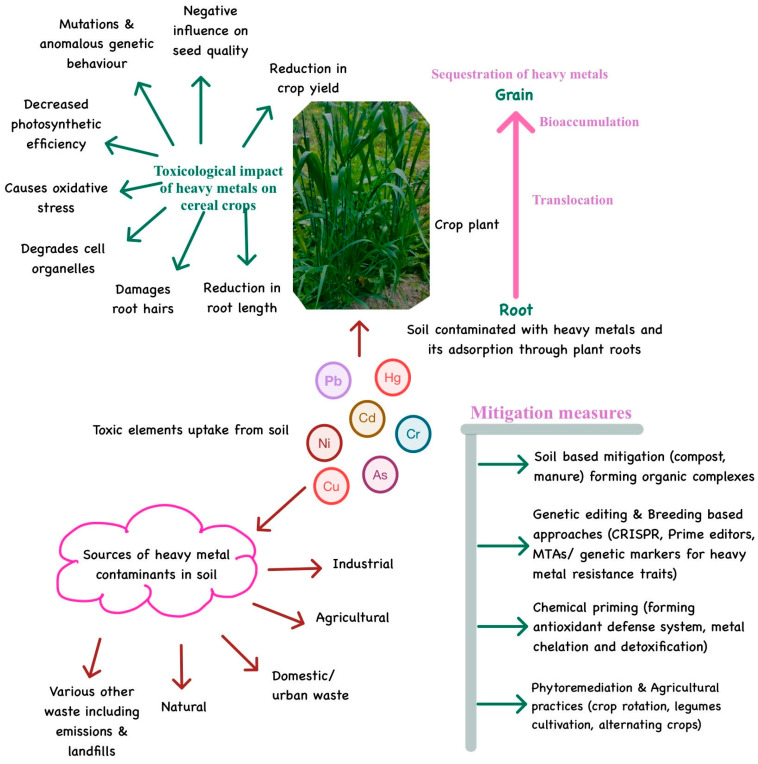
Sources of HMs contamination in soil, their uptake by plant roots, translocation in various plant parts, and sequestration in the edible grain. The higher HM concentration has an adverse effect on plant growth and quality, and its presence in the food grain further intoxicates the food chain. Mitigation of these HMs through several modes helps in eradicating their toxicity from the food materials and ecosystem.

Crops absorb HMs like arsenic and cadmium from contaminated soils, but uptake varies by species [68,69] and soil conditions such as organic content and pH [70,71,72,73]. For example, barley and rice tend to accumulate more metals than corn [74,75,76,77]. After absorption through root systems, metals move to different parts of the plant through phloem and then sequester into the grain, bringing several toxic impacts (Figure 1). Long-term exposure to these metals through staple foods like wheat can cause severe health issues, including cardiovascular diseases, cancer, and organ damage, highlighting the need for careful monitoring and management [78,79]. Acknowledging how HMs impact crops is a way to improve farming and sustainability. The toxicity of heavy metals in cereal crops depends on factors like organic content, soil pH, metal levels, and exposure duration (Table 1). These variables influence how heavy metals affect plant growth, highlighting the challenges faced in growing productive and safe cereals.

Several complex physiological and molecular processes influence the absorption, transport, and accumulation of heavy metals in plants. Root epidermal and cortical cells subsequently absorb the accessible metal ions via a variety of membrane transporters and channels, which are frequently shared with vital nutrients like Fe, Zn, and Mn. After being absorbed, metals are moved to aerial tissues through the xylem and phloem, where they may be stored, detoxified, or integrated into cellular structures. To understand how plants, especially wheat, a model species in heavy metal studies and a major cereal crop, manage metal homeostasis and deal with metal-induced stress under contaminated soil conditions, it is essential to comprehend these coordinated systems.

### 4.1. Cadmium Uptake and Transport Pathways in Wheat

Cd is a highly toxic soil pollutant, and it primarily originates from sewage sludge disposal, pesticides, fungicides, and the use of phosphorus-based fertilizers, i.e., industrial, and agricultural manufacturing. Cd has the potential to inflict extensive harm to root systems of plants and influence normal growth and development of plants. Cd also has the tendency to easily accumulate in crops and, in doing so, may also enter the food chain and become a hazard to human health [95]. Therefore, it is important to understand the mechanism and pathway of transport of cadmium from soil to roots to grains to humans in the food chain.

#### 4.1.1. Root Absorption of Cadmium

Among other things mentioned in Table 1, soil acidification mainly increases the bioavailability of Cd to plants, and root exudates make it more soluble. Both Cd^2+^ and Cd chelates are forms of cadmium that are present in soil solutions [96]. In plants, cadmium can move through the apoplastic and symplastic pathways (more complex due to the role of transmembrane transport proteins) in the leaves, stems, and roots [96,97]. Cd can reach the root cells through various transporters, which move different forms of Cd. For example, the natural resistance-associated macrophage proteins (NRAMP), like the AtNRAMP6; the zinc/iron-regulated transporter-like protein (ZIP), such as the *TcZIP4*/*TcZNT1* transporter; and the low-affinity calcium transporters are responsible for the transport of cadmium in Cd^2+^ form. Cd chelates reach the roots through yellow stripe 1-like (YSL) proteins. Cation channels are also involved in the transport of Cd to root cells. Table 2 depicts various channels associated with the entry of Cd into the roots.

#### 4.1.2. Translocation of Cadmium to the Xylem

Cadmium may likely enter the xylem through the symplastic transport and possibly through the apoplastic transport under intense exposure. Through all the barriers from the surface of the root to the root cortex, which includes apoplasmic barriers, such as the Casparian strip of the endodermis, metal ions move into the symplast and are carried to the stele and xylem elements [103,104]. Apoplastic routes allow solutes to move along the extracellular fluid and the gaseous interstitial spaces between and among cell walls, whereas symplastic routes involve solutes and water moving intracellularly, moving from cell to cell through tubular structures called plasmodesmata [104]. Regardless of the method, the most important stage for Cd transport is loading into the root xylem [104]. The apoplastic and symplastic pathways get regulated at certain locations in the root cortex, where cells in charge of loading Cd into root xylem are present [104]. Now the translocation of Cd is related to its retention in the roots along with proper loading into the xylem vessels. Retention is done via Cd-chelating molecules (phytochelatins), apoplastic barriers, and vacuolar sequestration [104,105].

#### 4.1.3. Systemic Movement of Cadmium to Shoots and Grains

There are three mechanisms responsible for metal transport from the root to the stem after metal uptake by the root symplast: metal sequestration by root cells, symplastic transport to the stele, and the delivery of metals into the xylem [104,106]. The process of loading of stem xylem is stringently regulated and mediated by membrane transport proteins yet to be characterized [104]. In hyperaccumulators, the coordination between metals and low molecular mass chelators increases the transpiration-mediated transport of these metals to the shoot, while in non-hyperaccumulating conditions, the increased cation exchange capacity of xylem cell walls arrests further metal ion transport [104]. Either after remobilization from leaves or after root uptake, xylem loading, and fast accumulation at the shoot base, Cd enters and exits growing grains directly through the phloem [104,107]. Xylem-to-phloem transfer is an important process in Cd uptake by leaves and grains [104,108]. Heavy metal ATPases (HMAs) play a crucial role in the translocation of Cd/Zn between the plant root and the shoots and can power the transport of heavy metals along the membranes [95]. These critical membrane-bound proteins transport metals across membranes with the help of the energy derived from the hydrolysis of ATP [109]. The phloem is the primary entry point for Cd into grains. An unknown 13 kDa protein and SH-compounds in the phloem sap may be the sites of Cd binding [98]. The metal trafficking takes place within each plant cell, regulating the concentrations of these molecules inside the specified range of physiology for each organelle, and the metal delivery to the proteins requiring them [97,110]. The type of cells in which these metals are deposited varies depending on the metal type and plant species. In response, plants show a range of defense mechanisms to manage the cadmium toxicity once it has reached the cells [12,104]. Table 3 discusses the effect of cadmium toxicity observed on cereals.

### 4.2. Toxicological Effects of HMs on Cereals

#### 4.2.1. Cadmium Toxicity

Cadmium (Cd^2+^) has high solubility in water, high mobility in soil colloids, high bioavailability in soil, and high translocability in plants and shows high lethal toxicity in plants [18]. In plants, cadmium competes with essential nutrients, disrupting physiological function, and damages key processes like water uptake and photosynthesis, which ultimately results in reduced growth and poor crop productivity [26,80]. Cadmium, being a carcinogenic and toxic metal, harms plant growth as well as human health [26].

#### 4.2.2. Chromium Toxicity

Chromium (Cr^3+^, Cr^6+^) has moderate solubility in water, moderate mobility in soil colloids, moderate bioavailability in soil, restricted translocability in plants, and shows moderately lethal toxicity in plants [18]. Toxicity symptoms induced by Cr exposure include: (i) wilting, (ii) chlorosis of leaves, and (iii) reduced shoot and root growth [76]. Upon invasion of plant tissue by Cr, it disturbs the structure of lamella and affects the growth as well as yield of *Triticum aestivum* [120]. The occurrence of chromium toxicity reduces the active reaction centers of photosystem II, reduces the rate of electron transport, and changes the heterogeneity of photosystem II [76,120]. Cr modifies the activity of enzymes and initiates the creation of reactive oxygen species (ROS), resulting in oxidative damage [121], which in turn interferes with the synthesis of lipids and the function of membranes, resulting in the oxidation of proteins and nucleic acids, causing damage to cellular components and, in certain situations, cell death [122]. Additionally, Cr, when accumulated in higher amounts, has been reported [123] to have a severe impact on the germination of seeds and growth of shoots and roots, which impacts the total yield and biomass [121]. Physiologically, it reduces water potential, nutrient uptake, and transpiration [10].

#### 4.2.3. Lead Toxicity

Lead (Pb^2+^) has low solubility in water, poor mobility in soil colloids, limited bioavailability in soil, restricted translocability in plants, and moderately lethal toxicity in plants [18]. Even at low doses, Pb toxic exposure is detrimental to plants, impeding normal plant growth and lowering crop production and output [10]. Reduced nutrient absorption and deactivated cell membrane permeability are clear signs of Pb poisoning in plants [10]. Lead, being a key heavy metal contaminant, can inhibit a range of enzymes and metabolic processes critical to chlorophyll biosynthesis [80]. A progressive yellowing of plant leaves is the result of lead-induced chlorosis interfering with the delicate balance of chlorophyll production. Chlorosis typically starts in mature cereal leaves, as HMs are taken up from the soil into the roots and subsequently moved upwards to the leaves [80]. With increasing levels of HMs in the leaves, chlorophyll levels decline, resulting in a progressive yellowing of the leaf tissue. Under severe HM poisoning, the condition of chlorosis can evolve into necrosis, where yellow leaves wilt and die [80,111]. Necrosis in cereals has serious implications for plant productivity and health [80]. Necrotic tissue loses its function, leading to decreased photosynthetic activity, water transport, and nutrient uptake [80]. Decreased functional photosynthetic tissue lowers carbohydrate content for grain formation, resulting in shriveled and poorly developed grains, ultimately influencing crop yield. Therefore, necrosis is a serious implication of heavy metal toxicity in cereals, which is the killing of plant tissues through oxidative stress and cell injury [80,117]. A detailed insight into the effect of lead toxicity is discussed in Table 4.

#### 4.2.4. Mercury Toxicity

Mercury (Hg) is another important environmental contaminant that remains in terrestrial soils and, therefore, is a huge worldwide concern [124]. Mercury (Hg^2+^) has low solubility in water, low mobility in soil colloids, moderate bioavailability in soil, limited translocability in plants, and shows moderately lethal toxicity in plants [18]. Hg occurs mostly in solid form, with the ionic form (Hg^2+^) being the most prevalent in agricultural soil matrices [125]. Plant biological system-Hg interaction is of extreme importance, considering the fact that mercury has been used in the past as a seed disinfectant, as well as to produce fertilizers and herbicides [125]. After interaction with plants, mercury has been reported to induce the formation of reactive oxygen species (ROS) like hydroxyl radicals, superoxide radicals, and hydrogen peroxide (H_2_O_2_) [124,126]. In toxicity, mercury exerts substantial inhibitory effects on root elongation, seed germination, and coleoptile and hypocotyl elongation in wheat compared to other HMs [126,127].

#### 4.2.5. Arsenic Toxicity

Arsenic (As^3+^, As^5+^) has high solubility in water, high mobility in soil colloids, high bioavailability in soil, high translocability in plants, and shows lethal toxicity in plants [18]. It is a highly hazardous element whose toxicity varies depending on the plant species [15,16,80]. For example, in wheat, it has been found to inflict various negative morphological and physiological changes such as decreased biomass of roots and stems, decreased number of spikes per plant, wilting of leaf margins, stunted roots and stems, and necrosis ultimately [15,80]. Whereas, in the case of Zea mays, it is known to decrease the chlorophyll content, and in rice its toxicity manifests in the form of increased lipid peroxidation, superoxide dismutase (SOD), and ascorbate peroxidase (APX) activity [13,20]. Other toxic symptoms due to arsenic include reduction in seed germination, dry matter production [18], crop yield, seedling height, leaf area, chlorosis, wilting, premature shedding of leaves, shortening of plant height, and reduction in the number and size of nodules.

## 5. Mechanistic Insights into HM-Induced Growth Constraints in Plants

Heavy metals (HMs) are mostly absorbed by plants through their roots from the soil solution, where they are found as ionic species. They can move through various cellular compartments with the help of a variety of transporter proteins and ion channels, such as ATP-binding cassette transporters, HM ATPases, and cation diffusion facilitators [127,128]. In addition to disrupting water balance, nutrient absorption, and mineral transport to aerial plant parts, heavy metals (HMs) that adversely affect root development ultimately impede overall growth, biomass accumulation, and productivity [18]. Additionally, when the internal concentrations of HMs exceed the plant’s tolerance limit, HMs harm vital macromolecules like proteins, lipids, carbohydrates, and nucleic acids as well as the structure and function of the organelles, including mitochondria, chloroplasts, nuclei, and vacuoles [129,130,131,132]. It has been demonstrated that elevated HM levels change the ultrastructure of chloroplasts, lower chlorophyll a/b ratios, interfere with the manufacture of photosynthetic pigments, and suppress the activity of both catalytic and non-catalytic proteins that are essential to plant growth and metabolism [18]. According to published research, the reactivity and concentration of heavy metals (HMs) in leaves determine how they affect photosynthetic machinery, which in turn impacts the basic mechanisms of photochemistry during the light-dependent stage of photosynthesis, electron transport, and the activities of photosynthetic enzymes like RuBisCO [18,133,134,135]. An overview of the principal mechanisms of heavy metal-induced growth inhibition and the corresponding adaptive responses is presented in Table 5, providing a comprehensive depiction of how plants perceive and mitigate heavy metal toxicity.

Heavy metal affects the structural and functional dynamics of plant stomata in addition to these internal physiological and biochemical disturbances. Numerous investigations of heavy metal-induced plant stomatal closure have been carried out, and the effects of heavy metals on various plants vary. Black gram, tobacco, and soybean plants can all have their stomata closed by lead [154,155,156]. *Brassica juncea*, rice, cowpea, *Pennisetum* sp., and *Hordeum vulgare* can all have their stomata closed by cadmium [157,158,159]. Zn can cause cowpea plants’ stomata to close [160]; Hg can cause spruce stomata to close [156]. These findings imply that stomatal closure, which is probably one of the compensatory mechanisms by which plants react to heavy metal stress, might result from varying heavy metal exposures in various plant species. Another significant marker of heavy metal stress is stomatal density [156]. Heavy metals have been demonstrated to alter plant stomatal density in a variety of ways. For instance, it has been demonstrated that plants under Cd stress have lower stomatal density [161]. Water hyacinth leaves with low concentrations of lead have been shown to have more stomata, while leaves with high concentrations of lead have fewer stomata [156,162]. In pea plants, Cd causes the guard cells’ radius to diminish, their length to decrease, and their width to rise [163,164]. Like this, Pb causes soybean plants’ stomatal guard cells to shrink in diameter, which results in the guard cell plastids producing a lot of starch grains and plastid globules [156,165]. Rice guard cells have been reported to be damaged severely due to Cd and Pb buildup [166,167]. It disrupts the stomatal function and damages the plant reproductive system [167]. Acknowledging the defense mechanisms that plants employ to defend themselves from the impact of various heavy metal toxicities is important for developing metal-tolerant crops and improving phytoremediation for sustainable agriculture.

## 6. Bioavailability and Bioaccumulation of Heavy Metals

Bioavailability is the strength of HMs in soil that is available for uptake by the plants or organisms [168]. Cereals are capable of absorbing heavy metals into their grains, thereby serving as vectors for human exposure [168,169]. Heavy metal bioavailability in cereals is a process that starts at the root-soil interface. Through their roots, cereals take in water and nutrients from the soil; heavy metals are not an exception.

Bioavailability is affected by numerous factors such as the chemical form of metals, interaction between nutrients, and interindividual differences [170,171]. Wheat, for example, has high cadmium adsorption capacity, which constitutes a serious issue where wheat forms a high percentage of the diet. Cadmium ionic species (Cd^2+^) are very bioavailable, while precipitated or complexed metal species would most likely have limited availability to plant roots [172]. Lead is deposited in outer regions of grains of lead-contaminated soils. It is absorbed by root-cereal crops mainly by active uptake processes [80]. Transport proteins are involved in the uptake of lead ions into root cells and also in the transport of necessary minerals like magnesium (Mg) and calcium (Ca) into root cells at the same time. Lead can go to above-ground plant parts, including leaves, grains, and stems, after being absorbed by the roots [80]. For less toxic and less mobile Cr(III), transport proteins embedded within the root cell membranes are said to be crucial in the uptake process. The transporters are assumed to allow the influx of Cr(III) ions into root cells. Cr(VI), being more soluble, is also linked with increased toxicity. Whilst the entire Cr(VI) uptake mechanism is known, it is believed to entail the uptake of chromate ions (CrO_4_^2−^) by particular anion transporters present in the membranes of the root cells. Translocation of chromium ions into other plant structures, such as leaves, grains, and stems, is controlled by plant physiological processes as well as by the specific chemical form of chromium [80,173].

The bioaccumulation characteristic of heavy metals tends to produce more harmful impacts on human health at low exposure levels [174]. Bioaccumulation is the mechanism by which living things, including animals and plants, absorb and store substances, in this case HMs, in amounts higher than in their surrounding environment. The chemical structure, also known as speciation, of heavy metals is a key factor in their bioaccumulation in soil systems [80]. Some of these metals are easier to incorporate into plants than others. Cereals, for instance, are often more accessible to heavy metals in soluble or exchangeable forms than in insoluble or complexed forms [80]. Some types of wheat have been found to be able to sequester lead and cadmium, particularly in the grain. Some types of wheat, however, can accumulate to varying degrees. The degree of bioaccumulation varies according to different cultivars. Heavy metals can cause oxidative stress, inhibit enzyme activity, and interfere with cellular processes through bioaccumulation, which can eventually cause several health problems in the long run [175,176].

## 7. Approaches to Control HM Bioaccumulation in Agroecosystems

The protection of agricultural viability and food safety requires measures to stop heavy metal accumulation in cereal crops that grow in the fields. Scientists have developed diverse methods to minimize the availability of the most persistent and dangerous heavy metals like cadmium, arsenic, chromium, and lead in cereal crops through modifications in both soil management and plant genetics [80,177]. These methods present promising strategies to either alleviate or mitigate the toxicity in plants (Figure 1), and in alignment with these initiatives, many nations have set regulatory limits for HMs in cultivation soils; for example, in India, Cd, Pb, and Cu have a limit of 3–6 mg/kg, 250–500 mg/kg, and 135–270 mg/kg, respectively; Sweden has set the limit of Cd and Pb at 0.4 mg/kg and 80 mg/kg, respectively; the UK has a limit of 8 mg/kg for Hg and 20 mg/kg for As, whereas Japan has a limit of 15 mg/kg for As and 125 mg/kg for Cu in the agriculture soil [178].

### 7.1. Soil-Based Mitigation Approaches

One of the most widely employed remedies in the mitigation field is soil amendments. Liming is an effective way to raise soil pH levels by applying calcium carbonate, which reduces the solubility and mobility of hazardous metals [71,167]. HMs in the soil become immobile when organic materials, such as compost and manure, are added because they form permanent organic material complexes. When cereals are grown in such soils, they show reduced metal uptake, which results in better crop quality and also protects human health. Supplements lower crop metal absorption rates through microbial support and structural improvement of organic matter. In addition to having metal-binding qualities that restrict their availability to plants, biochar, a carbon-based substance derived from biomass pyrolysis, shows promise in enhancing soil quality [179,180] but remains expensive and less accessible to small farmers. Numerous other techniques, such as applying selenium, applying boron, and subsurface drip irrigation, have also been utilized to lessen Cd buildup in crops and reduce the harmful effects of Cd on human health [181,182]. Regardless of their efficiency, soil-based amendments generally exhibit substantial variability across soil types, and long-term field data remain sparse. Many applications also risk disrupting soil chemistry or nutrient balance, potentially impacting beneficial microorganisms. In their entirety, these approaches are practical for short-term mitigation but require site-specific optimization for reliable performance in practical agriculture systems. In the future, the cost-effective and ecologically friendly methods paired with new technologies might boost the sustainability and efficiency of soil amendment.

### 7.2. Genetic and Breeding-Based Strategies

Genetic techniques, which aim to produce plants that reduce their capacity to absorb heavy metals and prevent them from entering their tissues, provide support for soil-based products. Through breeding programs and genetic testing, scientists discovered wheat varieties that naturally deposit less cadmium in their grains [183]. Crop enhancements aimed at micronutrient-related characteristics can be effectively facilitated by molecular breeding. GWAS (genome-wide association studies) have found marker-trait associations (MTAs) linked to micronutrients and other characteristics in wheat. This has led to the identification of genomic hotspots (the regions of interest on wheat chromosomes) [184] that may be markers, QTLs, or genes for desired attributes [185]. Cadmium-safe cultivars have previously been screened in durum wheat and sunflower [186]. Also, to lessen the harmful effects of chromium on both humans and plants, breeding activities targeted at creating crop types with high chromium resistance or tolerance are crucial. For example, regular corn cultivars do not exhibit excessive metal buildup, while sweet corn cultivars do [186]. Similarly, ZS 758 and Zheda 622 were cultivars of *B. napus* that accumulate chromium at low and high levels, respectively [187]. The new cultivars provide farmers with a practical solution for growing in polluted areas without compromising their agricultural output or yield possibilities [188]. The most cutting-edge and effective technique in contemporary agriculture is the use of the CRISPR/Cas system to increase crop resistance to Cr stress. While its growth in heavy metal stress tolerance is still in the experimental stage, applications of CRISPR/*Cas9*-related technologies are currently being used to edit the genomes of several crop plants to tolerate various biotic and abiotic stresses [189,190,191,192]. Breeding metal-resistant genotypes provides a low-cost, long-lasting solution once cultivars are accepted. However, the development process is long, resource-intensive, and complicated due to the environmental adaptability of cultivars. CRISPR-based methods offer high precision but face high initial costs and regulatory hurdles and have limited real-world application, highlighting the need for rigorous multi-environment trials and clearer biosafety networks.

### 7.3. Chemical Priming for Enhanced Metal Tolerance

An alternative strategy to these methods is the use of chemical priming as a method for enhancing plant resistance to metal-induced oxidative stress. Chemicals such as ABA, glutathione, cysteine, sulphur and melatonin have been observed to improve antioxidant defense systems, thus lessening Cr-induced damage [193,194]. Other compounds (e.g., MTs and H_2_S) induce metal chelation and detoxification involving the upregulation of stress response genes [195,196,197]. Moreover, 5-aminolevulinic acid (ALA), nitric oxide (NO), taurine, and mannitol would enhance photosynthesis, osmolyte regulation, and ROS scavenging under Cr stress [197,198,199,200]. In particular, glutathione is a key player, as it forms Cr–GSH complexes, offering lower metal mobility and protection of chlorophyll structure [201,202]. A brief outline of the crucial priming agents that are used and their mechanisms and impact on Cr stress tolerance is discussed in Table 6. Chemical priming is promising but limited by dose sensitivity, inconsistent field performance, and risks of phytotoxicity. Most findings come from controlled experiments, so field applicability and long-term ecological impacts remain uncertain, underscoring the need for clearer cost–benefit validation.

### 7.4. Monitoring and Regulatory Framework

Soil testing on a regular basis is fundamental for guiding interventions and choosing suitable crops for cultivation. Effective methods for monitoring allow for swift action and compliance with food safety standards, as national and international regulations set strict limits on heavy metals in cereals [80]. Enforcement through routine inspections also helps protect public health and maintain consumer trust in agricultural products [80,81]. Despite being vital, monitoring frameworks frequently suffer from limited infrastructure, irregular testing frequency, and gaps between policy and field-level compliance. Strengthening institutional capability and implementing real-time affordable sensor technologies could bridge these essential gaps.

### 7.5. Agronomic and Phytoremediation Practices

Agronomic methods like crop rotation and the inclusion of legumes play an essential role in reducing heavy metal buildup in agricultural soils. Farmers can limit the accumulation of metal over time by alternating cereal crops with non-cereal crops [211]. The use of hyperaccumulator species (like *Helianthus annuus* and *Brassica juncea*) for phytoremediation is another practical strategy, helping to extract metals from contaminated fields before cereals are planted [212,213,214]. Hyperaccumulators can remove metals but also pose issues such as biomass disposal and inconsistent uptake rates. These practices work best when combined with other interventions, underscoring the importance of a multi-layered, landscape-level management approach. Sustainable environmental remediation can be aided by the identification of appropriate cereal species and the optimization of growth conditions to enhance metal removal from polluted soils.

## 8. Conclusions

HMs are non-decomposable elements. The pollution from such elements as Cd, Cr, Pb, and Hg is a serious challenge to agricultural sustainability and food security, resulting from present human activity, leading to hazardous effects on crop productivity and the safety of consumers. This study emphasizes how important it is to understand how the presence of heavy metals in soils affects cereals. In-depth studies of the molecular, physiological, and ecological elements are essential for promoting innovation and creating workable solutions. Embracing the challenges posed by heavy metal pollution with proactive research and collaboration will equip us to develop a sustainable and secure future for cereal crops amidst the altering environmental backdrop.

A complicated and urgent problem for global food safety and environmental health is the effect of HM contamination in soil on cereal crops. This study has examined several topics pertaining to HM contamination in soil and how it affects cereals, highlighting important issues that call for in-depth research and creative solutions. Developing successful methods to reduce metal toxicity requires an understanding of the complex mechanism of HM uptake, transport, and sequestration in cereal crops. In-depth investigations into these mechanisms will enable us to exploit their potential for developing metal-tolerant cereal variants.

Symptoms of heavy metal poisoning, ranging from chlorosis and stunted growth to necrosis and water stress, demonstrate the substantial harm sustained by cereal crops. There is an urgent need for thorough research on plant physiology under metal stress due to the complex effects of HMs, as toxicity from HMs disrupts plant physiological processes, reducing photosynthesis and nutrient uptake, and damages DNA, causing symptoms such as stunted growth of the plant and poor grain quality. These symptoms highlight how important it is to investigate the underlying causes of cereals’ susceptibility to heavy metal poisoning. The degree of impact is shaped by soil characteristics, pH, organic matter content, plant types, and environmental factors, so a thorough grasp of efficient mitigation techniques is required. Plants utilize adaptive defenses—including metal sequestration and hormonal modulation—but comprehensive solutions require integrating soil, plant, and microbial strategies. Breeding, biotechnological innovations, soil amendments, and agronomic practices can limit metal accumulation in crops and restore soil health.

Field-scale validation of integrated or hybrid technologies is still limited, especially under variable climates and land-use patterns. There is a need to optimize these combined systems for multi-metal and multi-pollutant scenarios and to understand trade-offs such as slow remediation rates, plant toxicity, and impacts on soil structure and biodiversity. The development of metal-tolerant grain varieties through genetic and breeding procedures holds considerable potential for sustainable agriculture in metal-contaminated regions. Additionally, studying the potential of soil amendments and agronomic methods, such as liming and crop rotation, will benefit in reducing heavy metal bioavailability and toxicity in soil. This information then serves as the basis for developing focused mitigation plans that protect the production of cereals, adopt sustainable farming methods, and guarantee the nutritious value of cereals. As we venture beyond the existing state of the art, the dedication to innovation resonates as a beacon of hope for a resilient and secure future in cereal agriculture and global nutritional safety.

## Figures and Tables

**Table 1:** Factors Influencing Heavy Metal Uptake and Toxicity.

Factors	Features	Impact on HM Toxicity	References
Soil pH	Soil acidity/alkalinity affects the metal solubility. Acidic soils (pH < 7) increase solubility and bioavailability of metals like Cd, Al, Mn, etc. Alkaline soil reduces the solubility by precipitating metals.	Acidic soil increases metal uptake; alkaline soil reduces uptake but may cause nutrient deficiencies.	[80,81,82,83]
Soil organic matter	Decomposed plant and animal residues bind with metal ions (chelation). Humic and fulvic acids form stable complexes with metals. Supports beneficial microorganisms like mycorrhizae.	Reduces heavy metal mobility and uptake; improves soil structure and microbial health.	[80,81,84]
Soil texture	Texture (proportions of sand, silt, and clay) affects retention/release of metals. Clay soils adsorb metals strongly; sandy soils have low absorption, causing higher mobility.	Clay limits uptake via immobilization; sandy soils increase uptake due to leaching and mobility.	[80,85,86]
Plant species & varieties	Various species differ in heavy metal tolerance and detoxification. Examples: Barley and rye show higher tolerance. Low-cadmium rice varieties bred to limit Cd in grains. Root hair and exudates also play roles.	Tolerant varieties limit translocation; breeding can reduce grain contamination.	[80,87,88]
Soil metal concentration	Higher metal concentration increases uptake and toxicity. Exceeding toxicity threshold affects plant growth and food safety. Interactions between metals (synergistic effects) can intensify toxicity.	Elevated metal levels cause tissue damage and grain contamination; metal synergy worsens toxicity.	[80,89,90]
Temperature	High temperatures increase metal solubility and uptake by crops. Heatwaves enhance mobility of metals like Cd and Pb.	Increased risk of uptake and toxicity during high-temperature periods.	[80,91]
Humidity	High humidity may cause waterlogging and increase metal diffusion. Low humidity reduces water uptake, intensifying toxicity.	Waterlogged soils promote uptake; dry conditions exacerbate stress from existing heavy metal presence.	[80,92,93]
Water availability	Influences dilution or concentration of heavy metals in soil solutions. Adequate water reduces toxicity; drought increases risk.	Water scarcity heightens metal uptake; proper irrigation reduces availability of toxic metals.	[80,94]

**Table 2:** Transporters and Channels Involved in Cadmium Entry into Plant Roots.

Transporter/Channel	Function	Specificity/Substrate	Additional Description	References
AtIRT1	Plasma membrane metal transporter	Broad specificity for divalent metals (e.g., Fe^2+^, Zn^2+^, Cd^2+^)	Present in the outer root layer; absorbs metals from the soil	[98,99]
TcZNT1/TcZIP4	Zn transporter; low-affinity Cd uptake	High-affinity for Zn; low-affinity for Cd	Mediates Cd and Zn uptake when expressed in roots	[98]
OsNRAMP1/OsNRAMP5	Cd and Fe influx transporter	Fe^2+^, Cd^2+^	Plasma membrane localized; involved in Cd uptake	[98,100]
AtNRAMP6	Intracellular metal transporter	Cd^2+^	Functions inside the cell rather than at plasma membrane	[98]
TaLCT1	Influx cation transporter	Ca^2+^, Cd^2+^, K^+^	Broad substrate specificity; Cd transport inhibited by high Ca^2+^ or Mg^2+^	[98]
DACCs (Depolarization-activated Ca^2+^ channels)	Ca^2+^ influx channel	Non-selective for cations (including Cd^2+^)	Activated at −80 mV; unstable and appears infrequently	[98,101]
HACCs (Hyperpolarization-activated Ca^2+^ channels)	Ca^2+^ influx; guard cell signalling	Non-selective (includes Cd^2+^)	Involved in response to ABA, light, and elicitors	[98]
VICCs (Voltage-independent Ca^2+^ channels)	Ca^2+^ and Cd^2+^ influx	Non-selective	Likely overlap with DACCs and HACCs functions	[98]
YSL (Yellow Stripe-Like)	Transport of nicotinamide (NA)-metal chelates	NA–Fe, NA–Cd complexes	Oligopeptide transporter; induced under Fe deficiency	[98,102]

**Table 3:** Heavy Metal—Cadmium Toxicity.

Category	Effect of Cadmium Toxicity	Mechanism/Detail	References
Chlorosis	Yellowing of leaves	Disrupts chlorophyll synthesis by inhibiting enzymes like δ-aminolaevulinic acid dehydratase (ALAD) and protoporphyrinogen oxidase (PPO)	[80,111,112]
Stunted Growth	Reduced plant size and growth	Impairs root elongation and nutrient uptake, and interferes with growth hormone (auxins and gibberellin) synthesis	[80,113]
Reduced Root Growth	Inhibited root elongation and branching	Causes ROS accumulation in roots, disrupts cell division and elongation	[80,114]
Nutrient Uptake Interference	Leads to deficiencies in essential elements like zinc	Competes with zinc for uptake, reducing zinc availability	[80,114]
Leaf Deformities	Twisting, curling, and irregular leaf shapes	Interferes with gibberellin biosynthesis and causes oxidative stress	[80,115]
Reduced Flowering	Delayed flowering	Disrupts cytokinin signalling	[80,116]
Reduced Fruit Development	Smaller and malformed fruits	Competes with zinc, impacting zinc-dependent processes essential for fruit development	[80]
Necrosis	Formation of necrotic lesions and tissue death	Induces ROS accumulation, causing oxidative damage to proteins, lipids, and DNA	[80,117]
Water Stress	Wilting and reduced water uptake	Restricts root elongation, disrupts root cell membranes, and affects water transport mechanisms	[80,118,119]

**Table 4:** Heavy Metal—Lead Toxicity.

Category	Effect of Lead Toxicity	Mechanism/Detail	References
Chlorosis	Yellowing of leaves	Inhibits chlorophyll biosynthesis by disrupting enzymes and metabolic pathways	[80,111]
Stunted Growth	Reduced plant height and biomass	Interferes with elongation in roots and shoots and cell division, and disrupts nutrient/water uptake	[80,113]
Hormonal Disruption	Impaired growth regulation	Inhibits synthesis of growth hormones like auxins and gibberellins	[80]
Reduced Flowering	Decreased flower development and elongation of flower stalks	Inhibits gibberellin biosynthesis	[80,116]
Necrosis	Formation of necrotic lesions in leaves and tissues	Leads to ROS production, oxidative stress, and subsequent cell death	[80,117]

**Table 5:** Overview of Key Mechanisms of Plant Growth Inhibition by Heavy Metals.

Mechanism	Physiological/Molecular Effects	Heavy Metals Involved	Effects/Observation	References
DNA metabolism disruption	Causes DNA strand breaks, chromosomal aberrations, and inhibition of replication and repair enzymes; leads to genomic instability.	Cr, Cd, Pb, As, Hg	Arsenic inhibits poly-(ADP-ribose) polymerase-1; Cd and Pb induce double-strand breaks in *Vicia faba*; Hg binds covalently to DNA, causing sister chromatid exchange and mitotic disruptions.	[136,137,138,139]
Altered gene expression	Modifies expression of metal transporters (HMA, ZIP, NRAMP, ABC), signalling genes (MAPKs), and transcription factors; disrupts metabolic and defense gene regulation.	Cd, Zn, Hg, Cu, Pb	Cd interferes with Zn-finger TFs; barley overexpresses dehydration-related TFs under Cd and Hg stress; gene overexpression enhances metal uptake and phytoremediation potential.	[140,141,142,143]
Hormonal deregulation	Disrupts balance of growth and stress hormones (auxins, gibberellins, cytokinin, ABA, JA); alters signalling pathways affecting growth and defense.	Cd, Pb, Hg, Cu, Zn, Ni	Exogenous kinetin reduces Cd toxicity (*Pisum sativum*); GA_3_ alleviates Pb/Cd stress (*Vicia faba*, *Lupinus albus*); IAA and SA restore antioxidant activity in wheat; BSs mitigate Cd stress in tomato; ABA accumulation restricts metal translocation but inhibits growth.	[144,145,146,147,148]
Inhibition of soil microorganism	Reduces microbial biomass, diversity, and enzymatic activity essential for nutrient cycling; disturbs rhizosphere balance and soil fertility.	Zn, Cu, Pb, Cd, Hg	Inhibition of CO_2_ evolution due to impaired microbial respiration; decline in dehydrogenase activity and basal respiration; molecular analysis (16S/18S rRNA) reveals shifts in microbial community structure.	[149,150,151,152,153]
Overall impact on plant growth	Combined disruption of genetic stability, hormonal signalling, and soil microbial support leads to stunted growth, reduced photosynthesis, and poor yield quality.	Majority of heavy metals	Integrative stress effects on plant physiology, metabolism, and soil–plant–microbe interactions.	[18,147,150,152,153]

**Table 6:** Chemical Priming Agents and Their Role in Alleviating Cr Toxicity in Plants.

Priming Agent/Compound	Mode of Action/Mechanism	Plant Species Studied	Overall Impact on Cr Stress	References
ABA, Glutathione (GSH), Cysteine, Sulphur, Melatonin	Enhance detoxification processes, stimulate antioxidant enzyme systems, and limit Cr uptake	Various crops	Reduced oxidative stress and improved tolerance to Cr toxicity	[10,193,203]
Metallothioneins (MTs)	Chelate and immobilize Cr ions via thiol-rich ligands; upregulation of MT-related genes under stress	*Brassica napus*	Enhanced Cr detoxification and protection of cellular components	[10,195]
Hydrogen Sulphide (H_2_S)	Boosts antioxidant activity, upregulates MT genes, increases chlorophyll and thiol content, and promotes Cr-binding peptide synthesis	*B. napus*, *Barley*, *Arabidopsis*	Reduced lipid peroxidation, enhanced photosynthesis, and improved metal tolerance	[10,197,204,205]
5-Aminolevulinic Acid (ALA)	Stimulates chlorophyll synthesis, improves metabolism, and decreases Cr accumulation	*B. napus*	Enhanced growth and photosynthetic efficiency under Cr exposure	[10,196]
Taurine	Protects lipid membranes, enhances ROS scavenging, improves nutrient assimilation and osmolyte accumulation	*Triticum aestivum*	Increased biomass, membrane stability, and stress tolerance	[10,199]
Mannitol (M)	Acts as an osmoprotectant, decreases Cr translocation, and activates antioxidant enzymes	*Triticum aestivum*	Lowered Cr content and improved photosynthetic pigment levels	[10,200]
Glutathione (GSH)	Forms Cr–GSH complexes, neutralizes ROS via the ASA–GSH cycle, and limits Cr translocation	*Glycine max*	Maintained chlorophyll, higher biomass, and effective detoxification	[10,202,206]
Indole Acetic Acid (IAA)	Modulates antioxidant enzymes and hormonal signalling to minimize oxidative injury	*Pisum sativum*	Reduced ROS accumulation and improved stress resistance	[10,207,208]
Jasmonic Acid (JA)	Strengthens antioxidant and glyoxalase systems, maintains Ca^2+^ balance, and limits Cr uptake	*Brassica parachinensis*, *P. sativum*	Improved mineral homeostasis and lower Cr accumulation	[10,209,210]

## Data Availability

No new data were created or analyzed in this study. Data sharing is not applicable to this article.

## Abbreviations

HM	Heavy Metal
NRAMP	Natural Resistance-Associated Macrophage Protein
YSL	Yellow Stripe Like
IRT	Iron-regulated transporter
DACC	Depolarization-activated Ca^2+^ channels
WHO	World Health Organization
IAA	Indole acetic acid
JA	Jasmonic Acid
ALA	δ-aminolaevulinic acid
ABA	Abscisic acid
HACC	Hyperpolarization-activated Ca^2+^ channels
VICC	Voltage-independent Ca^2+^ channels
ZNT	Zinc Transporter
ZIP	Zn/Iron-regulated protein
LCT	Low-affinity Cation Transporter
GWAS	Genome-Wide Association Studies
MTA	Marker Trait Association
